# Platelet-Rich Fibrin in Bone Regenerative Strategies in Orthodontics: A Systematic Review

**DOI:** 10.3390/ma13081866

**Published:** 2020-04-16

**Authors:** Inês Francisco, Maria Helena Fernandes, Francisco Vale

**Affiliations:** 1 Institute of Orthodontics, Faculty of Medicine of the University of Coimbra, 3000-075 Coimbra, Portugal; ines70.francisco@gmail.com; 2 Faculty of Dental Medicine, University of Porto, 4200-393 Porto, Portugal; 3 LAQV/REQUIMTE, U. Porto, 4160-007 Porto, Portugal

**Keywords:** orthodontics, bone regeneration, platelet rich fibrin, platelet concentrate

## Abstract

Preservation of the alveolar bone is a determinant in the outcome of orthodontic treatment. Alveolar bone defects or a decrease of their height and width may occur due to common reasons such as inflammation, tooth extraction, or cleft lip and palate. The aim of this systematic review was to investigate and appraise the quality of the most up to date available evidence regarding the applications and effects of platelet-rich fibrin (PRF) in orthodontics. This study was carried out according to preferred reporting items for systematic reviews and meta-analyses guidelines using the following databases: Medline via PubMed, Cochrane Library, Web of Science Core Collection and EMBASE. The qualitative assessment of the included studies was performed using Cochrane Risk of Bias tool and ROBINS-I guidelines. Results: From a total of 489 studies, nine studies were selected. The majority of the included studies demonstrate that autogenous anterior iliac graft with PRF had a higher amount of newly formed bone. Furthermore, this review also suggests that the application of platelet derivatives in the extraction socket can accelerate orthodontic tooth movement. Despite the limitations in the included studies, this systematic review suggested that PRF can improve alveolar cleft reconstruction and orthodontic tooth movement.

## 1. Introduction

Regenerative therapy in oro-dental and maxillo-facial defects is challenging because the oral cavity has several tissues with distinct cell populations (ectodermal and mesodermal), making regenerative procedures more complex [1]. Bone and soft tissue regeneration may be indicated for managing defects subsequent from several conditions, such as congenital defects (cleft lip and palate), alveolar bone resorption, periodontal defects (recession coverage and furcation defects), cystic cavities, bone infection (osteomyelitis), and traumatic bone destruction [1,2,3,4]. Nowadays, the current clinical approaches have several limitations, namely limited self-renewal capacity and/or limited donor supply, risk of immune response, operative time, and costs and donor site morbidity. As a consequence, new biomaterials have been developed to modulate inflammation and enhance the healing process [5].

Platelet derivatives are increasingly used in regenerative dentistry, particularly in implantology, oral surgery, and periodontology. Platelets, 2–3 µm blood corpuscles, are cytoplasm fragments from the megakaryocytes in the bone marrow that then enter the circulation. Following tissue injury, activated platelets have a key role in soft and hard tissue regeneration. Platelet concentrates release a variety of cytokines and growth factors that promote the regenerative capacity of periosteum and improve bone and tissue healing and regeneration. Choukroun et al. reported that the platelet-rich fibrin (PRF) improves tissue repair and regeneration. PRF is prepared from centrifuged autologous blood with no addition of bovine thrombin or anticoagulants [6]. During blood centrifugation, two processes occur: (1) blood coagulation and (2) separation of blood elements due to the centrifugation force. Subsequently, three distinct layers are formed: platelet-poor plasma (top), PRF (middle zone), and red blood cells (bottom) [7].

This fibrin matrix contains platelets, leukocytes, growth factors and cytokines, such as interleukin (IL)-1β, IL-4, and IL-6, transforming growth factor-beta1 (TGF-β1), platelet-derived growth factor (PDGF), and vascular endothelial growth factor (VEGF) [6,7]. These factors can promote the proliferation/differentiation pathways of osteoblasts, endothelial cells, chondrocytes, and various sources of fibroblasts, which can stimulate the regenerative capacity of periosteum and enhance bone and tissue repair and regeneration [8]. Furthermore, the fibrous structure of PRF acts as a three-dimensional fibrin scaffold for cell migration [9]. Thus, PRF may be used with bone substitutes, which allows wound sealing, hemostasis, and improves bone maturation and graft stabilization. Furthermore, PRF membrane can be used for guided bone regeneration [2].

Tissue regeneration is a new emerging approach in orthodontics because a high percentage of patients need both regeneration and orthodontic treatment. Orthodontic treatment can be performed on children, young adults, and adults. All of these patients may need regenerative approaches due to different indications (e.g., children with cleft lip and palate who need closure of alveolar cleft; older patients who need an orthodontic treatment due to bone defect as a result of tooth loss). Moreover, the application of mechanical force on the teeth affects the periodontal ligament and the alveolar bone, which allows orthodontic tooth movement (OTM) [10]. Thus, a change in support structures may interfere with orthodontic success. Therefore, the use of PRF can improve orthodontic treatment results, since it promotes a biological response involving a minimally invasive procedure. Moreover, PRF is completely autologous, requires minimal biochemical handling of blood, provides release of growth factors over time, and it is easy to prepare and cost effective [11]. During recent years, clinical applications and effects of PRF in regenerative dentistry have been reviewed, but studies on the application of PRF in orthodontics are sparse.

### Objective

The purpose of this review was to systematically investigate and appraise the quality of the most up to date available evidence from human studies regarding the applications and effects of PRF in orthodontics. 

## 2. Materials and Methods 

### 2.1. Protocol

This systematic review was designed and reported according to the Preferred Reporting Items for Systematic Reviews and Meta-Analyses (PRISMA) guidelines and Cochrane guidelines for Systematic Reviews [12,13]. The PICO (Population, Intervention, Comparison and Outcome) research question was: “What is the application and effects of Platelet-Rich Fibrin in orthodontic treatment?”

The protocol for this systematic review was registered on PROSPERO and waiting for registration number. 

### 2.2. Eligibility Criteria

Table 1 describes the PICO research question.

### 2.3. Search Strategy and Study Selection

Four electronic databases (Medline via PubMed, Cochrane Library, Web of Science Core Collection, and EMBASE) were searched until 16 December 2019 independently by two reviewers (I.F., F.V.). 

To conduct the research, a combination of medical subject headings (MeSH) with relevant free text words was used in each database. Table A1 summarises the search strategies. The following language filters were applied: English, Portuguese, and Spanish. Furthermore, no restrictions of publication date were applied. A manual search of the references lists of the retrieved full text articles was also conducted.

Articles were screened based on the titles and abstracts according to the eligibility criteria by two independent reviewers, in duplicate. Subsequently, full texts were screened for potential inclusion and disagreements were resolved through mediation with a third reviewer (M.F.). 

The following inclusion criteria were considered: (i) randomised controlled trials (RCTs), controlled clinical trials (CCTs) and cohort studies; (ii) studies in humans; (iii) orthodontic patients; (iv) reported hard tissue reconstruction or rate of tooth movement as outcome (v) the study should evaluate the applications and effects of PRF on orthodontics. The exclusion criteria were as follows: considered: (i) non-clinical studies and all other research types (for example, editorials, textbooks, and technical reports); (ii) edentulous patients; (iii) animal studies; (iv) case reports or descriptive studies; (v) repeated publications; (vi) studies with missing data.

### 2.4. Data Extraction

For data extraction, a standard form was developed. The information that was extracted from each article included: field of study, first author and year of publication, study design, aim of study, number of participants in experimental and control group, PRF protocol, results and main conclusions. In the case of uncertainty or discrepancies between the reviewers (I.F., F.V.), a third reviewer was consulted (M.F.).

### 2.5. Risk of Bias

Two reviewers assessed the methodological quality of recruited studies independently. For both RCTs and CCTs, the Cochrane Risk of Bias tool was used [14]. The domains evaluated were: (1) random sequence generation, (2) allocation concealment, (3) blinding of participants and personnel, (4) blinding of outcome assessment, (5) incomplete outcome data, (6) selective reporting, and (7) other bias. Risk of bias is detailed in Table A2. The overall risk of bias of individual studies was categorized as low (if all domains were considered as having a low risk of bias), unclear risk (if one or more domains were at unclear risk of bias) and high (if at least one domain was at high risk of bias).

For cohort studies, the qualitative assessment of the selected studies was performed using the risk of bias in non-randomized studies of interventions (ROBINS-I) assessment tool [15]. The domains evaluated were: (1) Bias due to confounding; (2) Bias in selection of participants into the study; (3) Bias in classification of interventions; (4) Bias due to deviations from intended intervention; (5) Bias due to missing data; (6) Bias in measurement of outcomes; (7) Bias in selection of the reported result. This information is summarized in Table A3. The overall risk of bias of individual studies was categorized as low (if all domains were considered as having a low risk of bias), moderate (if low or moderate risk of bias for all domains), serious (if at least one domain was at serious risk of bias), critical (if at least one domain was at critical risk of bias) and no information (if no clear indication that the study is at serious or critical risk of bias and there is a lack of information in one or more key domains of bias). 

## 3. Results

### 3.1. Selection of the Studies

A total of 489 studies were identified from electronic databases. After the removal of duplicates using EndNote reference management software (Clarivate Analytics, Philadelphia, PA, USA), 426 records were screened based on the titles and abstracts. In the case of uncertainty or discrepancies, the article was included. Subsequently, 25 studies were reviewed and evaluated according to the eligible criteria. The final sample included nine studies. 

The included studies were single centre studies. All included articles were parallel two-arm trials [2,16,17,18,19,20,21], except the two studies that were split-mouth trials [22,23]. 

**Figure 1:**
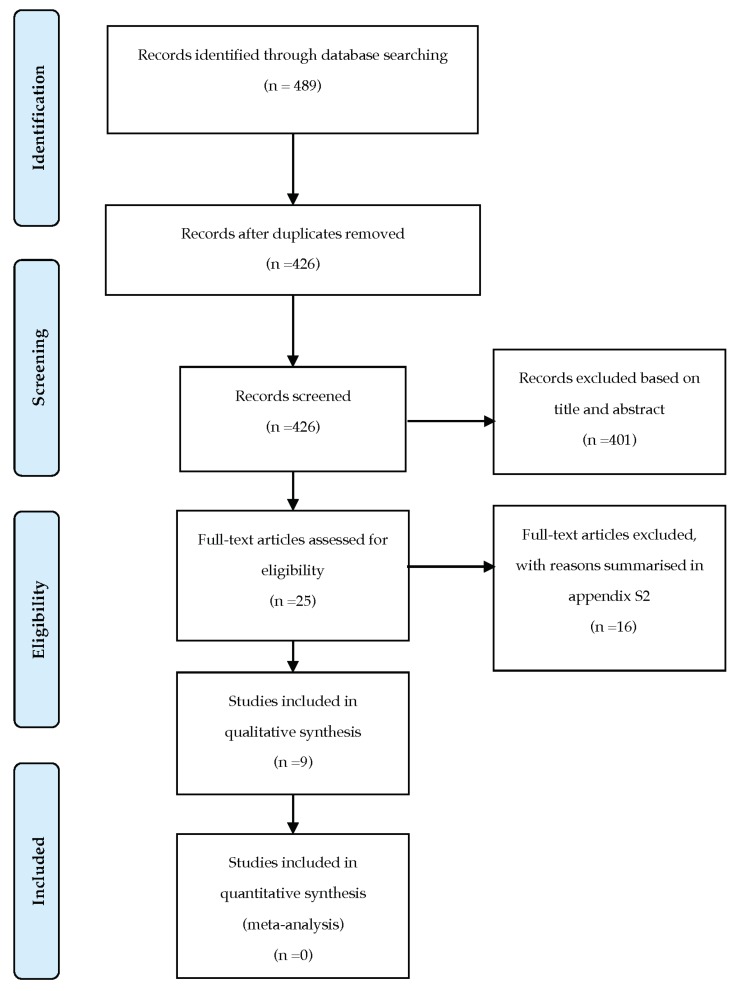
PRISMA flow chart.

The identification, screening, and eligibility process is summarised in the PRISMA flow chart (Figure 1).

The reasons for the excluded records are summarised in Table A4 [24,25,26,27,28,29,30,31,32,33,34,35,36,37,38,39]. 

### 3.2. Characteristics of Included Studies

The general information of included studies is summarised in Table 2 and Table 3. The studies involved a total of 175 patients aged 6–51 years, and were recent, undertaken between 2016 and 2019. Six studies [2,16,17,18,19,20] investigated the efficacy of PRF in maxillary alveolar cleft reconstruction. Four of them compared autogenous bone graft from anterior iliac crest with and without PRF. The other two studies [17,18] investigated the combination of PRF with other approaches: (1) allogenic bone material and chin symphysis bone [17], and (2) autologous bone marrow mononuclear cells (BMMNCs) combined with nanohydroxyapatite [18]. In the six studies, the cone beam computer tomography (CBCT) or computed tomography scan (CT) was used as an assessment tool to measure outcome. 

Three articles investigated tooth movement and post-orthodontic stability [21,22,23]. Two studies evaluated the amount of OTM by decreasing the horizontal linear distance between the mid-marginal ridges of the adjacent teeth [22,23]. Only one study considered clinical parameters and patient feedback to evaluate pain, post-surgical inflammation, and infection [21]. 

### 3.3. Risk of Bias

**Figure 2:**
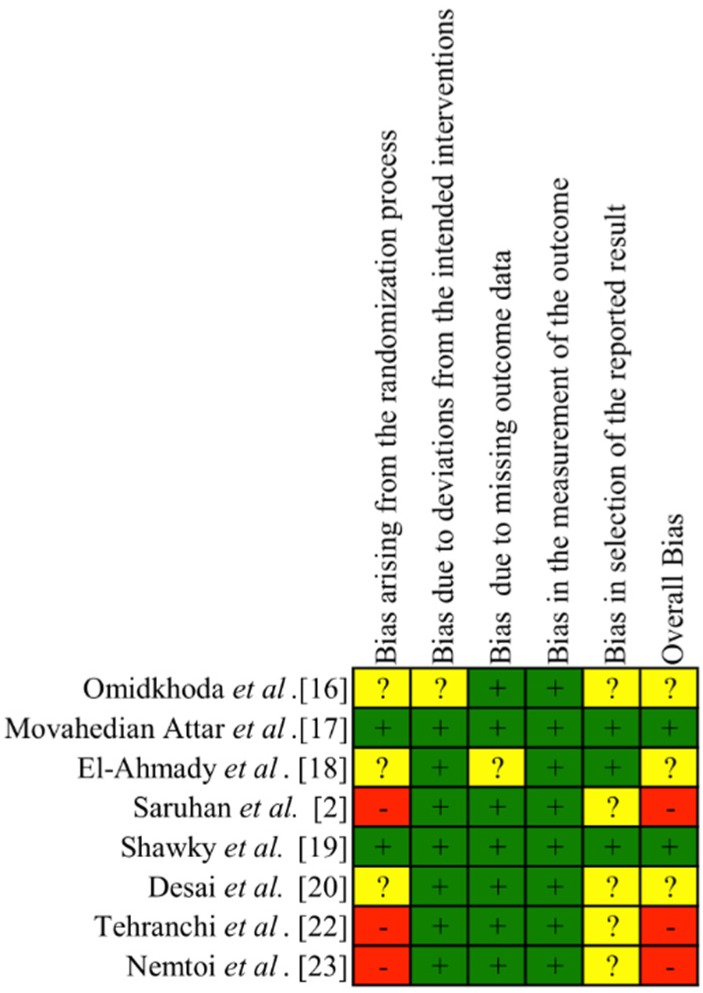
Risk of bias of the RCTs and CCTs studies. + low risk of bias. ? unclear risk of bias. – high risk of bias.

The results of the quality assessment of the RCTs and CCTs studies are summarized in Figure 2. Three studies were judged as having high risk of bias, mostly due to deviations from the randomization process [2,22,23]. Two trials were judged as low risk of bias [17,19]. The remaining studies were considered as unclear risk due to deviations from the randomization process [16,18,20] and bias in selection of the reported results [16,20].

Cohort study was considered as a moderate risk of bias due to deviations from the selection of participants into the study, the measurement of outcomes and the selection of the reported results [21]. The remaining domains showed a low risk of bias. The limited number of trials did not allow risk of bias assessment across studies.

### 3.4. Quantitative Synthesis of the Results

The heterogeneous interventions and treatment performed in the included articles did not allow a quantitative synthesis of the results. Furthermore, the heterogeneity in the design and methodologies precludes the quantitative analysis of results. 

### 3.5. Results of Included studies

There was a range of different PRF protocol preparation in the included studies. Five studies [2,16,17,19,21] adjusted centrifugation to 3000 rpm for 10 min, while others used 3000 rpm for 20 min [18], 2900 rpm for 10 min [20], and 2700 rpm for 12 min [22,23]. 

The nine studies were grouped under two categories: (1) alveolar cleft reconstruction and (2) tooth movement and post-orthodontic stability.

#### 3.5.1. Alveolar Cleft Reconstruction

All the works that evaluated alveolar cleft reconstruction comprised a control group in which patients went through maxillary alveolar cleft reconstruction with autogenous anterior iliac crest bone graft [2,16,17,18,19]. Four studies compared control group with autogenous anterior iliac graft only with PRF [2,16,19,20]. Omidkhoda et al. showed that PRF combined with autogenous bone did not have a significant increase in the thickness, height, and density of alveolar bone graft [16]. The other three studies suggested that experimental group had higher amount of newly formed bone [2,19,20]. 

Saruhan et al. reported that the mean percentage of newly formed bone was 68.21 ± 10.80% and 64.62 ± 9.49% in experimental and control group respectively [2]. Desai et al. evaluated vertical bone height with a four-point scale (type 1—0%–25% resorption; type 2—25%–50%; type 3—50%–75%; type 4—75%–100%). In the experimental group, 18 patients had grade 1 resorption and two patients had grade 2 at nine months. In the control group, 12 patients had grade 1 resorption and eight patients had grade 2 at nine months [20]. Shawky et al. verified that the experimental group had higher percentage of newly formed bone (82.60%) compared with control group (68.38%). This study is the only one with statistically significant results assessing the percentage of newly formed bone [19].

Concerning the mean bone density, experimental group demonstrated lower values than control group with no statistically significant differences. Shawky et al. reported values of 384.03 HU and 360.82 HU, respectively for control and experimental groups, at six months follow-up [19]. Omidkhoda et al. also verified that the mean bone density was lower in experimental group (302.83 HU) than on control group (349.58) at three months after surgery [16]. 

The two studies that included other materials [17,18] have found that PRF combined with other regenerative materials was an appropriate graft material for reconstruction in alveolar clefts defects. Movahedian et al. evaluated the efficiency of the combination of bone graft from allogenic bone material, chin symphysis bone and leucocyte- and platelet-rich fibrin (L-PRF) and verified that the percentages of bone reconstruction were lower in experimental group (69.57 ± 10.13%) than in control group (73.86 ± 6.93%), without statistical differences between the two groups [17]. Otherwise, El-Ahmady et al. showed that 70% of the experimental group (using PRF with autologous BMMNCs and nanohydroxyapatite) presented bone tissue at the cementoenamel junction of the teeth next to the cleft covering at least 75% of both roots against 30% of the control group, at 12 months follow up [18]. 

The most frequent outcome measures were volumetric measurements and percentage ratios such as height, thickness and length. Four studies evaluated augmentation, bone reconstruction and graft ratios by comparing pre and postoperative 3D X-rays [2,17,19,20]. The evaluation of alveolar resorption or residual bone ratio and postoperative follow up was performed using 3D X-rays [16,18]. The follow–up ranged from 3 to 12 months across the studies. During the follow-up period, different outcomes were reported. Two studies did not verify complications (dehiscence, flap necrosis and infection or persistent oro-nasal fistula) [17,19], whereas another two studies identified persistent oro-nasal fistula in 30% of the control group [18] and dehiscence in four patients in the experimental group (n = 20) and eight patients in control group (n = 20) [19]. The other two included studies [2,16] did not report any information.

#### 3.5.2. Tooth Movement and Post-Orthodontic Stability

Two trials evaluated the effect of L-PRF or PRF on orthodontic tooth movement [22,23]. Both studies showed that application of these platelets’ derivatives in the extraction socket can accelerate orthodontic tooth movement (*p* = 0.006) [22,23], and it has been shown by Tehranchi et al. that there was no statistical difference between teeth in the maxillary and mandibular arch on OTM rate [22]. 

The study by Muñoz et al. was the only one that evaluated the effects of L-PRF considering inflammation and post-orthodontic stability. They demonstrated that, in a high percentage of patients (72.7%), edema resolution was set about day 4, and orthodontic stability was preserved for two or more years post-surgery in all patients [21].

## 4. Discussion

Alveolar cleft reconstruction and accelerated orthodontic tooth movement are matters of concern in contemporary orthodontics [28,40]. However, few studies were found assessing application of PRF on orthodontics compared to other dentistry areas.

All included articles in this systematic review studied the advantages of PRF in the orthodontics field. Although a quantitative report of the findings was not possible, qualitative systematic reviews still improve the current understanding and provide a critical appraisal of research relevant to regenerative orthodontics.

To evaluate the postoperative newly formed bone, 2D or 3D X-rays are required in addition to clinical assessment. Three-dimensional radiological studies had some benefits compared with two-dimensional ones, namely the three-dimensional location of the bone graft and the assessment of teeth eruption process on alveolar graft [41]. The advantages of CBCT over CT include the possibility to scan small regions for specify diagnosis, minimal scanning time (10–70 s), low radiation dose, and reduced image artefact [42]. In the present study, CBCT scan was used in five trials [2,16,17,18,23] and CT scan in two trials [19,20]. Due to its high performance, CBCT should be a standard treatment outcome method for the assessment of newly formed bone in further studies [28]. According to Thuaksuban et al., the remodelling process with cortical maturation occurs after six months, becoming stable until month 24 [43]. Therefore, CBCT should be carried out six months after the bone graft. Regarding the included studies, Omidkhoda et al. only evaluated a three month time-point [16], and thus their results should be carefully analysed as the remodelling process may not be completed.

Orthodontic treatment combined with surgical approaches is a common procedure in cleft lip and palate patients. In these patients, the treatment begins at birth and continues into adulthood usually requiring prosthesis in an anterior region or mesial movement of the posterior teeth to space closure of agenesis, mainly of the upper lateral incisor [44]. Alveolar cleft reconstruction with bone graft allows for an adequate volume of alveolar bone, which is fundamental for the dental movement in the maxillary aesthetic zone throughout the orthodontic treatment. Thus, the orthodontist can perform more stable and aesthetic treatments. In patients with alveolar cleft, autogenous iliac crest bone is the gold standard [45]. Autogenous bone graft is osteoconductive, osteoinductive and a source of osteogenic precursor cells [19]. However, new strategies, such as the use of PRF, have been advanced to speed up bone formation, reduce bone resorption and enhance soft tissue healing. PRF is a platelet concentrate without addition of thrombin or anticoagulants [6]. The physiologic polymerization in PRF allows the cytokines and growth factors to be stored and then slowly released, ensuring bioactive levels for a long time-period (up to 28 days) [46]. Besides protecting the surgical site, PRF membranes promote soft tissue healing functioning like a matrix to support neoangiogenesis, and migration of stem cells and osteoprogenitor cells into the graft [6,19]. In line with this, PRF decreases bone resorption and hasten wound healing in soft and hard tissues [11], which might contribute to the lower prevalence of complications during the follow-up period observed in the PRF group compared to the control group, reported by El-Ahmady et al. [18] and, also, to the increased new bone regeneration and better wound healing observed by Desai et al. [20]. These results are in line with other stating that PRF increased significantly root coverage [47,48].

Regarding orthodontic tooth movement, several non-invasive or invasive techniques have been proposed for accelerating this process. Most non-invasive techniques need more studies to prove their clinical effectiveness [49]. Invasive techniques appear to be more effective in promoting orthodontic tooth movement. The bone injury associated to the surgical procedure triggers a tissue reaction that enhances normal molecular and cellular events involved in tissue healing [50]. Somehow, the application of PRF mimics the surgical-induced healing capabilities, also inducing tissue regeneration. Being a physiologic polymerized fibrin matrix, PRF incorporates platelets, leucocytes, bioactive molecules and trapped circulating stem cells and progenitors to promote local tissue healing [6]. The two trials included in the present systematic review showed that the use of PRF or L-PRF in the extraction socket could accelerate OTM (*p* = 0.006), specifically in the beginning of orthodontic treatment (alignment and leveling) [22,23]. These results are in line with previous ones reporting that the application of several bioactive grafts can increase the bone maturation without interfering with the natural healing process [51]. However, cytokines and growth factors levels are maintained for a long time-period (up to 28 days). Liou demonstrated that the clinical effect of application of platelet derivatives could last 5–6 months with the faster rate of orthodontic movement in 2–4 months [52]. Although no conclusion of the potential effect of PRF on this process could be drawn based on these two trials, there was a trend that PRF has the ability to increase tooth movement. Thus, the application of PRF may shorten the orthodontic treatment time reducing associated costs, which nowadays is a concern in orthodontic patients, specifically in adults and patients with longer treatments such as those needing tooth extractions. Nevertheless, PRF has some disadvantages, namely the limited volume that can be produced and used, and tissue banks are impracticable, as it is specific to the donor and cannot be used as an allogenic graft tissue [6].

### 4.1. Limitations in this Review

The methodological and clinical heterogeneity among studies only allowed to qualitatively account the findings of this systematic review. Newly formed bone measurement tools were inconsistent across studies. Furthermore, most of the selected studies were classified as having a high or unclear risk of bias, which may decrease the certainty of the results. The heterogeneity of studies can be justified by the methodological differences across the studies, such as sample sizes, intervention protocols and follow-up times. Furthermore, several factors can affect local bone remodeling, namely age at surgery, width of the cleft defect, volume of grafted bone, and position of teeth on bone graft.

The authors recognize that the expertise of the clinician and support team, as well as the scientific proficiency of the all research group, influence the outcome evaluation. Some of the selected studies did not assess this factor, which should be considered when figuring out the results of this review.

### 4.2. Recommendations for Future Research

Given the inconsistent results presented in the limited literature, it is recommended to perform further research with standardized methodologies, a larger sample size, and longer follow-up to evaluate the applications and effects of PRF in the orthodontics field. Possible sources of bias should be controlled, such as randomization procedure, PRF protocols preparations, measurement tools of newly formed bone, and follow-up periods. Further studies should also investigate the cost–benefit analysis of using PRF in orthodontics for patients and clinicians.

## 5. Conclusions

Despite the limitations in the included studies, this systematic review suggested that PRF can improve alveolar cleft reconstruction. Concerning orthodontic tooth movement, the results highlight the positive effects of PRF, since it may shorten the orthodontic treatment time, thereby reducing associated costs.

Further, high-quality randomised controlled trials with identical methodologies, larger sample size, and longer follow-up periods are required.

## Figures and Tables

**Table 1:** Research question according to the PICO format.

Parameter	Assessment
Population (P)	Orthodontic Patients of any gender or age
Intervention (I)	Participants who underwent treatments approaches with the use of PRF with/without a combined biomaterial.
Comparison (C)	The control group consisted of participants that underwent treatments approaches without PRF.
Outcome (O)	Outcome were: - hard tissue reconstruction of alveolar bone—assessed by volume of the newly formed bone (measured in cubic centimeter or percentage of newly formed bone); - rate of tooth movement—assessed by the change in horizontal linear distance between the mid-marginal ridges of the adjacent teeth (measures in millimeters).

**Table 2:** Characteristics of included studies on alveolar cleft reconstruction.

Alveolar Cleft Reconstruction						
**Study**	Omidkhoda et al. [16]	Movahedian Attar et al. [17]	El-Ahmady et al. [18]	Saruhan et al. [2]	Shawky et al. [19]	Desai et al. [20]
**Year**	2018	2017	2018	2018	2016	2019
**Study design**	Parallel-group RCT	Parallel-group RCT	Parallel-group RCT	Parallel-group RCT	Parallel-group RCT	Parallel-group RCT
**Aim of study**	Efficacy of PRF in the quality and quantity of maxillary alveolar cleft repair.	Efficacy of (i) combination of symphysis bone, allograft and PRF, and (ii) iliac bone graft, in the regeneration of cleft defects.	Use of autologous BMMNCs combined with PRF and nanohydroxyapatite as an effective technique for alveolar cleft repair.	Effect of PRF in alveolar bone grafting using volumetric analysis.	Effect of PRF in the quality and quantity of unilateral maxillary alveolar cleft repair.	Efficacy of PRF for secondary alveolar bone grafting.
**Interventions**	Autogenous anterior iliac graft with PRF (n = 5)	Bone graft from allogenic bone material, chin symphysis bone and L-PRF (n = 10)	Autologous BMMNCs combined with nanohydroxyapatite and autologous PRF (n = 10)	Autogenous bone graft from anterior iliac crest with PRF (n = 17 alveolar cleft segment)	Autogenous bone graft from anterior iliac crest with PRF (n = 12)	Autogenous bone graft from anterior iliac crest with PRF (n = 20)
**Control**	Autogenous anterior iliac graft (n = 5)	Autogenous bone graft from anterior iliac crest (n = 10)	Autogenous bone graft from anterior iliac crest (n = 10)	Autogenous bone graft from anterior iliac crest (n = 14 alveolar cleft segment)	Autogenous bone graft from anterior iliac crest (n = 12)	Autogenous bone graft from anterior iliac crest (n = 20)
**Sample Size (females/males)**	10 (4/6)	20 (9/11)	20 (12/8)	31 alveolar cleft segments in 22 patients (13/9)	24 (8/16)	40 (19/21)
**Participant age (mean ± SD)**	9–12 (11.3 ± 0.83)	8–14 (9.7 ± 1.7)	8–15 (11.50 ± 7.55)	6–28 (17.71 ± 5.4)	9–14 (10.92 ± 2.75)	9–18 (15.29 ± 4.79)
**PRFProtocol**	3000 rpm, 10 min	3000 rpm, 10 min	3000 rpm, 20 min	3000 rpm, 10 min	3000 rpm, 10 min	2900 rpm, 10 min
**Outcome assessed**	CBCT images (Planmeca, Finland, 2009). Exposure parameters: field of view of 90 × 100 mm, voxel size of 200 µm, X-ray tube kilovoltage of 88 kVp, and 8 mA.	CBCT Images (Cranex 3D, Sordex, Helsinki, Finland). Exposure parameters: 0.5 mm scan thickness for axial cuts.	Panoramic radiographs and CBCT images. Pain was measured with a numerical scale score reporting pain intensity.	CBCT Images (NewTom FP, Quantitative Radiology, Verona, Italy). Exposure parameters: 0.5 mm scan thickness for axial cuts.	CT scan (Philips Brilliance 32 Slice.Cardiac MDCT, Philips Healthcare, city, Netherlands) of upper jaw. The axial cuts were 0.625 mm thick.	Orthopantomogram, upper occlusal view, and CT scan (KODAK 9000C and KODAK 9000C 3D extra oral Imaging System, 2016; Carestream Inc., New York, USA).
**Follow up**	3 months. CBCT images: immediate postoperative and 3 months after surgery.	12 months. CBCT images: before surgery and 12 months after. Clinical controls: 1week, 1, 3, 6 and 12 months.	12 months. CBCT images: 6 and 12 months after surgery. Clinical Controls: 1 day, 1 and 3 weeks, 6 and 12 months after surgery.	6 months. CBCT images: preoperative and 6 months after surgery. Clinical controls: every week during the first month; every month for the next 5 months.	6 months. CBCT images: preoperative and 6 months after surgery.	9 months. Radiographic assessment: preoperative; immediate, 3, 6 and 9 months postoperative.
**Conclusion**	PRF group did not have a significant increase in the thickness, height, and density of alveolar bone graft.	Averagely 69.5% of alveolar defects were regenerated with bone in experimental group and 73.8% on control group (*P* value = 0.156). Chin symphysis bone and allogenic bone material combined with L-PRF was an appropriate graft material.	Experimental group demonstrated 90% of complete alveolar bone union verses 70% in control group. Autologous BMMNCs in combination with autologous PRF and nanohydroxyapatite promote bone regeneration in alveolar clefts defects.	Postoperative newly formed bone volume was better in the experimental group (68.21%) than in control group (64.62%). Although, no statistically significant difference was found.	The mean amount and percentage of newly formed bone volume was higher in the experimental (0.78 cm^3^; 82.6%) than control group (0.62 cm^3^; 68.38%). Bone density does not increase, but the difference was not statistically significant.	PRF in combination with autogenous bone results in higher osteogenic effect which increases new bone regeneration and better wound healing.

RCT, randomized controlled trial. PRF, platelet-rich fibrin. SD, standard deviation. CBCT, cone beam computer tomography. L-PRF, leucocyte- and platelet-rich fibrin. BMMNCs, bone marrow mononuclear cells. CT, computed tomography scan.

**Table 3:** Characteristics of included studies on tooth movement and post-orthodontic stability.

Tooth Movement and Post-Orthodontic Stability			
**Study**	Muñoz et al. [21]	Tehranchi et al. [22]	Nemtoi et al. [23]
**Year**	2016	2018	2018
**Study design**	Cohort	Split mouth clinical trial RCT	Split mouth clinical trial CCT
**Aim of study**	Effect of L-PRF in PAOO concerning post-operative pain, inflammation, infection and post-orthodontic stability.	Effect of LPRF on OTM in extraction cases.	Efficacy of PRF in accelerating bone regeneration and orthodontic tooth movement.
**Interventions**	Wilcko’s modified PAOO technique combined with L-PRF	Extraction socket with LPRF (n = 15)	Extraction socket with LPRF (n = 20)
**Control**	NA	Extraction socket with secondary healing (n = 15)	Extraction socket with secondary healing (n = 20)
**Sample Size (females/males)**	11 (8/3)	Thirty extraction sockets in 8 patients (3/5)	Forty extraction sockets in 20 patients (11/9)
**Participant age (mean ± SD)**	15–51	12–25 (17.37 ± 12.48)	12–20 (16.43)
**PRF Protocol**	3000 rpm, 10 min	2700 rpm, 12 min	2700 rpm, 12 min
**Outcome assessed**	Clinical parameters and patient feedback were used to evaluate pain, post-surgical inflammation and infection.	OTM was measured by comparing the change in horizontal linear distance between the mid-marginal ridges of the adjacent teeth on a regular basis.	CBCT images (PlanmecaPromax 3D Mid, Planmeca OY, Helsinki, Finland). Exposure conditions: 90 kV, 12 mA, and exposure time of 18.3 s. OTM was measured by comparing the change in horizontal linear distance between the mid-marginal ridges of the adjacent teeth on a regular basis.
**Follow up**	Clinical evaluation: 1, 2, 4, 8 and 10 days post-operative.	16 weeks. OTM measurements: 2, 4, 6, 8, 10, 12, 14 and 16 weeks.	24 weeks. OTM measurements: before placement of PRF; 4, 8, 12, 16, 20 and 24 weeks after placement of PRF.
**Conclusion**	(1) No severe pain; (2) Edema resolution begun by day 4 with most patients (72.7%); (3) Orthodontic treatment average time was 9.3 months; (4) All cases maintained stability for at least 2 years.	LPRF group: decreased horizontal linear measurement between the mid marginal ridges of teeth (*p* = 0.006). Therefore, LPRF may accelerate OTM, particularly in extraction cases.	PRF group: decreased horizontal linear measurement between the mid marginal ridges of teeth. Therefore, LPRF may accelerate OTM, particularly in extraction cases.

RCT, randomized controlled trial. PRF, platelet-rich fibrin. SD, standard deviation. CBCT, cone beam computer tomography. L-PRF, leucocyte- and platelet-rich fibrin. PAOO, periodontally accelerated osteogenic orthodontics. OTM, orthodontic tooth movement. CCT, controlled clinical trial.

## Appendix A

**Table A1:** Search Strategy for each of the databases

Database	Search Equation
Medline (via PubMed)	(“Orthodontics”[Mesh] OR “Tooth Movement Techniques”[Mesh] OR “Orthodontic Brackets”[Mesh] OR “ Tooth Movement” OR “Accelerating Orthodontic” OR “Surgery, Oral”[Mesh] OR “Alveolar Bone Grafting”[Mesh] OR “Post extraction Socket” OR “Socket Preservation” OR “Guided Tissue Regeneration”[Mesh] OR “Bone Regeneration”[Mesh] OR “Tissue Scaffolds”[Mesh] OR “Bone Transplantation”[Mesh] OR “Bone Remodeling”[Mesh] OR “Bone Substitutes”[Mesh]) AND (“Platelet-Rich Fibrin”[Mesh] OR “Platelet Rich Fibrin” OR “Fibrin rich in growth factors” OR “Platelet concentrate” OR “PRF” OR “Second generation platelet rich fibrina” OR “osteoinductive biomaterials”)
Cochrane Library	(Mesh descriptor: [Orthodontics] OR Mesh descriptor: [Alveolar Bone Grafting] OR Mesh descriptor: [Bone Regeneration] OR tooth movement OR alveolar bone grafting) AND (Mesh descriptor: [Platelet-Rich Fibrin] OR PRF OR Platelet rich fibrin)
Web of Science Core Collection	TS = (platelet rich fibrin* OR platelet-rich fibrin * OR PRF* OR second generation platelet concentrate* OR platelet concentrate*) AND TS = (orthodontics* OR tooth movement* OR alveolar bone grafting* OR orthodontic brackets*)
EMBASE	(“Orthodontics” OR “Orthodontic tooth movement” OR “Orthodontic device” OR “Alveolar Bone Grafting”) AND (“Tissue regeneration” OR “bone regeneration”) AND (“Platelet-rich fibrin” OR “platelet AND concentrate”)

**Table A2:** Version 2 of the Cochrane tool for assessing risk of bias (RoB 2) [14].

Bias Domain and Signalling Question *	Response Options		
	Lower Risk of Bias	Higher Risk of Bias	Other
**Bias arising from the randomisation process**			
1.1 Was the allocation sequence random?	Y/PY	N/PN	NI
1.2 Was the allocation sequence concealed until participants were enrolled and assigned to interventions?	Y/PY	N/PN	NI
1.3 Did baseline differences between intervention groups suggest a problem with the randomisation process?	N/PN	Y/PY	NI
Risk-of-bias judgment (low/high/some concerns)			
Optional: What is the predicted direction of bias arising from the randomisation process?			
**Bias due to deviations from intended interventions**			
2.1 Were participants aware of their assigned intervention during the trial?	N/PN	Y/PY	NI
2.2 Were carers and people delivering the interventions aware of participants’ assigned intervention during the trial?	N/PN	Y/PY	NI
2.3 If Y/PY/NI to 2.1 or 2.2: Were there deviations from the intended intervention that arose because of the trial context?	N/PN	Y/PY	NA/NI
2.4 If Y/PY/NI to 2.3: Were these deviations likely to have affected the outcome?	N/PN	Y/PY	NA/NI
2.5 If Y/PY to 2.4: Were these deviations from intended intervention balanced between groups?	Y/PY	N/PN	NA/NI
2.6 Was an appropriate analysis used to estimate the effect of assignment to intervention?	Y/PY	N/PN	NI
2.7 If N/PN/NI to 2.6: Was there potential for a substantial impact (on the result) of the failure to analyse participants in the group to which they were randomised?	N/PN	Y/PY	NA/NI
Risk-of-bias judgment (low/high/some concerns)			
Optional: What is the predicted direction of bias due to deviations from intended interventions?			
**Bias due to missing outcome data**			
3.1 Were data for this outcome available for all, or nearly all, participants randomised?	Y/PY	N/PN	NI
3.2 If N/PN/NI to 3.1: Is there evidence that the result was not biased by missing outcome data?	Y/PY	N/PN	NA
3.3 If N/PN to 3.2: Could missingness in the outcome depend on its true value?	N/PN	Y/PY	NA/NI
3.4 If Y/PY/NI to 3.3: Is it likely that missingness in the outcome depended on its true value?	N/PN	Y/PY	NA/NI
Risk-of-bias judgment (low/high/some concerns)			
Optional: What is the predicted direction of bias due to missing outcome data?			
**Bias in measurement of the outcome**			
4.1 Was the method of measuring the outcome inappropriate?	N/PN	Y/PY	NI
4.2 Could measurement or ascertainment of the outcome have differed between intervention groups?	N/PN	Y/PY	NI
4.3 If N/PN/NI to 4.1 and 4.2: Were outcome assessors aware of the intervention received by study participants?	N/PN	Y/PY	NI
4.4 If Y/PY/NI to 4.3: Could assessment of the outcome have been influenced by knowledge of intervention received?	N/PN	Y/PY	NA/NI
4.5 If Y/PY/NI to 4.4: Is it likely that assessment of the outcome was influenced by knowledge of intervention received?	N/PN	Y/PY	NA/NI
Risk-of-bias judgment (low/high/some concerns)			
Optional: What is the predicted direction of bias in measurement of the outcome?			
**Bias in selection of the reported result**			
5.1 Were the data that produced this result analysed in accordance with a prespecified analysis plan that was finalised before unblinded outcome data were available for analysis?	Y/PY	N/PN	NI
Is the numerical result being assessed likely to have been selected, on the basis of the results, from:			
5.2 ... multiple eligible outcome measurements (e.g., scales, definitions, time points) within the outcome domain?	N/PN	Y/PY	NI
5.3 ... multiple eligible analyses of the data?	N/PN	Y/PY	NI
Risk-of-bias judgment (low/high/some concerns)			
Optional: What is the predicted direction bias due to selection of the reported results?			
**Overall bias**			
Risk-of-bias judgment (low/high/some concerns)			
Optional: What is the overall predicted direction of bias for this outcome?			
Y = yes; PY = probably yes; PN = probably no; N = no; NA = not applicable; NI = no information			

**Table A3:** Robins-I tool for assessing risk of bias for non-randomised studies [15].

Domain	Explanation
Pre-intervention	Risk of bias assessment is mainly distinct from assessments of randomised trials
Bias due to confounding	Baseline confounding occurs when one or more prognostic variables (factors that predict the outcome of interest) also predicts the intervention received at baseline. ROBINS-I can also address time-varying confounding, which occurs when individuals switch between the interventions being compared and when post-baseline prognostic factors affect the intervention received after baseline.
Bias in selection of participants into the study	When exclusion of some eligible participants, or the initial follow-up time of some participants, or some outcome events is related to both intervention and outcome, there will be an association between interventions and outcome even if the effects of the interventions are identical. This form of selection bias is distinct from confounding—A specific example is bias due to the inclusion of prevalent users, rather than new users, of an intervention.
At intervention	Risk of bias assessment is mainly distinct from assessments of randomised trials
Bias in classification of interventions	Bias introduced by either differential or non-differential misclassification of intervention status. Non-differential misclassification is unrelated to the outcome and will usually bias the estimated effect of intervention towards the null. Differential misclassification occurs when misclassification of intervention status is related to the outcome or the risk of the outcome, and is likely to lead to bias.
Post-intervention	Risk of bias assessment has substantial overlap with assessments of randomised trials
Bias due to deviations from intended interventions	Bias that arises when there are systematic differences between experimental intervention and comparator groups in the care provided, which represent a deviation from the intended intervention(s). Assessment of bias in this domain will depend on the type of effect of interest (either the effect of assignment to intervention or the effect of starting and adhering to intervention).
Bias due to missing data	Bias that arises when later follow-up is missing for individuals initially included and followed (such as differential loss to follow-up that is affected by prognostic factors); bias due to exclusion of individuals with missing information about intervention status or other variables such as confounders.
Bias in measurement of outcomes	Bias introduced by either differential or non-differential errors in measurement of outcome data. Such bias can arise when outcome assessors are aware of intervention status, if different methods are used to assess outcomes in different intervention groups, or if measurement errors are related to intervention status or effects.
Bias in selection of the reported result	Selective reporting of results in a way that depends on the findings and prevents the estimate from being included in a meta-analysis (or other synthesis).

**Table A4:** List of excluded studies.

Study	Reason for Exclusion
Alhasyimi et al. [24]	Animal study
Che et al. [25]	Review article
Dukka et al. [26]	Case Report
Janssen et al. [27]	Evaluates PRP and not PRF
Stasiak et al. [28]	Systematic review
Shetty et al. [29]	Trial registration
Subbalekha et al. [30]	Trial registration
Avinash et al. [31]	Trial registration
Tehranchi et al. [32]	Trial registration
Mazzone et al. [33]	Not orthodontic field
Shah et al. [34]	Technical note
Iskenderoglu et al. [35]	Case Report
Dimofte et al. [36]	Review article
Nadon et al. [37]	Evaluate a derivate of PRF
Aras et al. [38]	Case Report
Findik et al. [39]	Case Report
